# Over One Thousand Prescriptions

**Published:** 1892-12

**Authors:** 


**OVER ONE THOUSAND PRESCRIPTIONS OR FAVORITE FORMULÆ OF VARIOUS AUTHORS, TEACHERS AND PRACTICING PHYSICIANS.—Published by the Illustrated Medical Journal Co., Detroit, Mich. Price, $1.00.**

THIS little volume presents the bulk of the "favorite prescriptions" that have appeared in the columns of *Leonard's Illustrated Medical Journal* from time to time during the past five years. The index has been carefully prepared, with numerous cross-references, so as to put the large number of prescriptions contained therein at the immediate service of the user of the volume. Blank pages have been inserted to allow of the copying of any particular formulæ that may commend themselves. It will be found of inestimable value.

